# Beyond Angiography: Integrating Advanced Diagnostic Modalities in Coronary Stent Restenosis—a Systematic Review

**DOI:** 10.7150/ijms.119908

**Published:** 2026-03-25

**Authors:** Zihan Liu, Mingduo Zhang, Min Zhang, Xiantao Song, Dongfeng Zhang

**Affiliations:** 1 Capital Medical University, Beijing, China.; 2 Beijing Anzhen Hospital, Capital Medical University, Beijing, China.

**Keywords:** in-stent restenosis, fractional flow reserve, multimodal imaging integration

## Abstract

In-stent restenosis (ISR) continues to be a significant problem after percutaneous coronary intervention (PCI), negatively affecting patient care. This review offers a thorough examination of current ISR diagnostic methods - which combine anatomical and functional assessments with cutting-edge technologies - with holistic recommendations for ISR management, from optimized prevention during PCI to effective treatment.

Anatomically, coronary angiography (CAG) persists as the gold standard, while intravascular ultrasound (IVUS) and optical coherence tomography (OCT) enhance stent optimization and ISR detection through high-resolution imaging. Functionally, fractional flow reserve (FFR) and instantaneous wave-free ratio (iFR) quantify ischemic risk, whereas non-invasive techniques like Computed Tomography-derived Fractional Flow Reserve (CT-FFR) and quantitative flow ratio (QFR) are transforming clinical paradigms. Multimodal imaging fusion and artificial intelligence markedly improve diagnostic accuracy and efficiency. Biomarkers and genomics are valuable tools for assessing ISR risk. Future directions emphasize integrated anatomical-functional-molecular assessments and AI-driven personalized management to refine ISR care and patient prognosis.

## 1. Background

CHD is the leading cause of death from cardiovascular issues globally. Percutaneous coronary intervention (PCI) has become a standard treatment for CHD thanks to recent advancements in coronary intervention technologies. As a pivotal technique within PCI, coronary stent implantation markedly improves patient prognosis and therapeutic implications. The issue of In-stent Restenosis (ISR) remains a persistent challenge in the field of medicine. Systematic synthesis of recent advances in ISR research and optimization of diagnostic strategies is therefore of substantial clinical importance for guiding evidence-based decision-making.

ISR occurs when the artery narrows again after the stent is implanted, often due to excessive new tissue growth. ISR is angiographically characterized by a lumen narrowing of at least 50% of the vessel diameter associated with evidence of functional significance (ischemic symptoms or abnormal fractional flow reserve) or a luminal narrowing of at least 70% in the absence of ischemic symptoms within 5 mm proximal or distal of the implanted stent 1.

Advances in understanding ISR pathogenesis have refined diagnostic methodologies, while simultaneously laying a theoretical foundation for the prevention of ISR. Although coronary angiography (CAG) remains the diagnostic gold standard, intravascular ultrasound (IVUS) and optical coherence tomography (OCT) are increasingly prioritized for their high-resolution visualization of vessel wall pathology.

The clinical importance of ISR lies not only in the severity of anatomical stenosis, but also in the impact on myocardial ischemia. Functional evaluations critically assess the ischemic burden and prognostic implications of ISR lesions. Despite their clinical value, functional assessments face practical limitations in widespread adoption. Meanwhile, advances in biomarker research offer additional diagnostic insights.

Advances in medical imaging, artificial intelligence (AI), and deep learning are creating new, non-invasive ways to evaluate function. Computed tomography angiography-derived FFR (CT-FFR) facilitates rapid hemodynamic profiling of ISR lesions without invasive catheterization. AI-powered deep learning algorithms autonomously characterize ISR lesion morphology and synthesize clinical data to predict progression risks and hemodynamic impacts. Such multimodal, non-invasive diagnostic methods offer potential for streamlining procedures, lowering expenses, and enhancing accessibility, ultimately broadening patient benefits.

## 2. Anatomical Evaluation

### 2.1 Coronary Angiography (CAG)

CAG remains the gold standard for assessing coronary artery stenosis. However, its clinical application is constrained by its invasive nature, high cost, and procedure-related complications, including vascular endothelial injury and pseudoaneurysm formation.

Conventional CAG struggles to delineate complex lesion morphology due to the diverse nature of coronary disease, encompassing varying degrees of severity and spatial patterns 2. Future advancements will combine computer-assisted guidance with AI-powered systems to improve image recognition and registration, leading to more adaptable and effective image fusion techniques.

### 2.2 Coronary Computed Tomography Angiography (CTA)

CTA is a non-invasive imaging modality that provides detailed visualization of vascular walls and plaque characteristics, enabling the assessment of ISR severity and spatial distribution, while partially serving as an alternative to conventional CAG. Although CTA provides distinct advantages for diagnosing ISR, its accuracy can be affected by several factors, including stent artifacts, contrast concentration, and scan parameters 3,4. Therefore, a comprehensive diagnostic approach that integrates additional modalities is essential. The limited spatial resolution of CTA hinders its ability to accurately assess intra-arterial inflammation and makes comprehensive plaque characterization a lengthy process 5. Consequently, rapid quantitative identification of high-risk plaques and precise stratification of plaque-related risks remain significant clinical challenges.

Advancements in AI and deep learning have accelerated the development of real-time CTA-X-ray image fusion technology, demonstrating the potential for automated and intelligent image processing. A process of three-dimensional reconstruction of CTA data is followed by registration and integration with intraprocedural X-ray images to generate synthesized visualizations 6. In ISR interventions, this approach enhances visualization of in-stent structures and facilitates accurate assessment of stent expansion and neointimal distribution, potentially reducing procedural time, contrast load, and radiation exposure while improving intervention safety and efficacy. Furthermore, real-time visual navigation substantially improves anatomical precision and biomechanical compatibility during interventional procedures, methodically enhancing post-procedural safety outcomes 7.

Current image fusion techniques are often tied to specific company-made equipment and lack universality. Despite this, CTA-real-time X-ray image fusion technology has shown significant promise in interventional navigation by combining 3D vascular modeling with 2D fluoroscopic image spatial registration. However, due to the complexity of lesions, it still faces challenges in addressing position-related differences that affect accuracy and in enabling precise dynamic monitoring of disease progression 8.

### 2.3 Intravascular Ultrasound (IVUS)

IVUS utilizes distinct signal characteristics generated by ultrasound waves penetrating tissues to acquire internal coronary artery images via a miniature probe, enabling tissue composition analysis. As the gold standard for plaque assessment, IVUS demonstrates strong concordance with histopathological findings, differentiating arterial wall layers and identifying atherosclerotic plaque components 9.

Major adverse cardiovascular events (MACE) serve as the primary hard endpoint for evaluating clinical outcomes and the efficacy of therapeutic interventions in ISR. The IVUS-XPL study reported that patients meeting IVUS optimization criteria exhibited significantly lower rates of MACE within several years compared to non-optimized cases 10. In ISR, IVUS enables precise measurement of minimal stent area, quantification of neointimal hyperplasia volume, and detection of edge dissection—all of which inform the mechanism and optimal treatment of restenosis.

Despite its diagnostic utility, IVUS is constrained by several clinical limitations. First, it requires specialized operator expertise and entails high equipment and procedural costs, limiting widespread adoption. Second, IVUS exhibits relatively low spatial resolution—typically approximately 150 μm axially and 300 μm laterally—which restricts its ability to detect minute lesions, subtle plaque changes, and minor degrees of stent malapposition 11. Consequently, IVUS cannot accurately characterize the fine structural details of coronary plaques. Procedurally, standardized protocols for IVUS-guided stent optimization remain inconsistent, contributing to operator-dependent variability in stent selection, inflation pressure, and apposition assessment, thereby limiting procedural reproducibility 12. Furthermore, IVUS fails to adequately evaluate multifocal or diffuse coronary lesions, limiting its utility in diffuse ISR where multiple stenotic segments within a single stent require assessment and where determining the hemodynamically significant culprit segment is critical for targeted repeat intervention 13.

ISR is not a histologically homogeneous phenomenon; it encompasses both benign fibrotic neointimal hyperplasia and high-risk neoatherosclerosis characterized by lipid-rich necrotic cores, calcification, and thin-cap fibroatheroma—distinctions with important prognostic and therapeutic implications. Virtual histology intravascular ultrasound (VH-IVUS) utilizes a four-color coding system to classify distinct lesion types, demonstrating strong histopathological correlation in both native and stented coronary segments, is uniquely positioned to make this distinction *in vivo* 14. By integrating radiofrequency ultrasound backscatter data with amplitude information, VH-IVUS identifies specific plaque components and provides direct visualization of vessel wall pathology as a catheter-based intracoronary imaging modality. It allows detailed characterization of plaque composition, burden, distribution, and remodeling patterns, thereby enhancing diagnostic accuracy for borderline lesions 15. Consequently, VH-IVUS enhances the detection of vulnerable plaques, facilitates the prediction of MACE, and refines therapeutic decision-making. Furthermore, VH-IVUS serves as a critical diagnostic adjunct for minute lesions that elude characterization by conventional angiography and clinical risk predictors. The technique also enables evaluation of neointimal tissue components within ISR lesions and identifies morphological features predictive of ISR recurrence 16.

Integrated backscatter intravascular ultrasound (IB-IVUS), IVUS near-infrared spectroscopy (IVUS-NIRS), and IVUS with automated differential echogenicity (ADE) offer distinct advantages for vascular tissue characterization. Specifically, IB-IVUS quantifies tissue-specific backscatter signal properties, enabling reliable differentiation between fibrous tissue, lipid-rich plaques, and calcified lesions. This capability facilitates assessment of atherosclerotic plaque stability 17. Conversely, IVUS-NIRS represents a hybrid modality combining ultrasound-based structural imaging with near-infrared spectroscopic analysis. This integration permits comprehensive lesion assessment: IVUS characterizes vascular wall anatomy, while NIRS identifies lipid-rich and vulnerable plaques through chemical composition analysis. The approach is particularly valuable for ISR diagnosis, as it simultaneously visualizes neointimal hyperplasia within stents and characterizes its compositional features.

Moreover, ADE employs an automated algorithm to analyze echogenicity variations across tissue types. This enhances both the accuracy and efficiency of IVUS in classifying vascular lesion components 18. By reducing subjectivity inherent in manual interpretation, ADE improves IVUS reliability for detecting vulnerable plaques and informing optimal interventional strategies.

### 2.4 Optical Coherence Tomography (OCT)

A pivotal mechanism underlying ISR is suboptimal stent apposition. This condition induces hyperproliferation of vascular endothelial and smooth muscle cells, culminating in recurrent luminal stenosis. OCT directly visualizes stent-vessel wall interfaces, enabling precise identification of malapposition sites—including edge dissections, inter-strut tissue prolapse, and incomplete stent expansion 19. OCT has emerged as the reference standard for morphological characterization of ISR, providing detailed visualization of in-stent tissue that is essential for uproviding detailed coronary artery visualization and guiding phenotype-matched repeat intervention. It facilitates precise outlines of coronary anatomy, quantitative assessment of stenosis severity, and real-time guidance during PCI 20. Moreover, OCT effectively detects thrombogenic risk factors, including stent malapposition, under expansion, and nascent atherosclerosis, thereby informing targeted interventions to mitigate stent thrombosis 21. Compared with conventional CTA, OCT demonstrates superior performance in defining ISR morphology through enhanced visualization of neointimal hyperplasia characteristics, including thickness stratification, spatial distribution patterns, and tissue homogeneity, enabling refined classification and severity grading 22.

However, OCT exhibits several limitations in ISR assessment: its imaging depth is confined to the vascular intima and partial media, restricting comprehensive evaluation of deep vascular pathologies and providing inadequate data for complex deep-layer lesions. Similar to IVUS, OCT requires specialized equipment, trained operators, and complex workflows, resulting in elevated procedural costs. Imaging acquisition necessitates patient cooperation and contrast flushing for blood clearance, posing challenges in patients with renal insufficiency or contrast allergies. Furthermore, rigorous blood clearance during OCT procedures may induce transient ischemic symptoms such as chest pain or angina, increasing procedural risks and potentially complicating assessment in patients with severe ISR where even transient ischemia is poorly tolerated 23-25.

A summary of key clinical technologies pertaining to imaging evaluation can be found in Table 1.

## 3. Functional Evaluation

While imaging-based diagnostic methods effectively evaluate coronary artery stenosis severity and lesion morphology, they lack precision in assessing the hemodynamic relationship between vascular narrowing and myocardial ischemia. Hemodynamic functional evaluation techniques determine whether myocardial ischemia coexists within the vascular territory, thereby guiding clinical decisions regarding coronary artery disease intervention. A summary of key clinical technologies pertaining to multimodal functional evaluation can be found in Table 2.

### 3.1 Fractional Flow Reserve (FFR)

Fractional flow reserve (FFR), an invasive hemodynamic index, represents the ratio of maximum achievable myocardial blood flow distal to a coronary stenosis to the theoretical maximum flow in the same vessel under normal conditions. In 1993, Pijls et al. first reported the seminal work establishing FFR methodology 27. Previous studies demonstrated that an FFR threshold ≤0.80 reliably identifies myocardial ischemia associated with stenosis. The measurement protocol employs a 0.014-inch pressure wire to record distal coronary pressure, while proximal pressure is obtained via the guiding catheter. Vasodilators (e.g., adenosine or adenosine triphosphate) are administered during measurement to induce maximal hyperemia 28.

FFR reflects the relationship between coronary stenosis and myocardial perfusion, serving as the gold standard for evaluating the functional impact of coronary lesions. This technique provides hemodynamic data unaffected by heart rate, blood pressure, or acute hemodynamic changes, thus compensating for the anatomical limitations of CAG. When compared to CAG, FFR-guided PCI in patients with borderline lesions demonstrates significant clinical benefits, including reduced contrast usage and stent deployment, alongside improved patient outcomes and enhanced procedural safety 29. Decreased FFR values within ISR lesions correlate with elevated risks of MACE, establishing FFR as a critical prognostic indicator for refined risk stratification 30. The FAME3 trial identified post-PCI FFR as a significant predictor of target vessel failure at both vessel and patient levels, with lower post-PCI FFR values associated with higher revascularization rates 31.

Despite its clinical utility, FFR faces three principal limitations that are particularly pronounced in the assessment of ISR. First, its invasive nature carries inherent procedural risks, crossing the lesion with a pressure wire carries additional risk of dislodging fragile neointimal tissue or thrombus 32. Second, adenosine administration is contraindicated in certain populations; in ISR patients, who are frequently older and have multiple comorbidities, the prevalence of such contraindications is notably higher. Even in eligible populations, 12%-18% of patients exhibit adenosine resistance, necessitating dose escalation that increases gastrointestinal adverse effects, such as nausea and vomiting, without guaranteeing optimal measurement conditions 33. Third, microvascular dysfunction systematically compromises FFR accuracy by impairing maximal hyperemic response, leading to underestimation of ischemic severity. This is especially critical in ISR patients, many of whom have underlying diabetic or chronic inflammatory conditions associated with microvascular disease. A multicenter prospective study by Seitz et al. found that in patients with FFR values greater than 0.80, those with concomitant microcirculatory dysfunction had a significantly increased risk of MACE within two years 34. This suggests that relying solely on FFR may overlook the risks associated with microcirculatory abnormalities, necessitating combined assessment with coronary flow reserve. For ISR patients with preserved FFR, unrecognized microvascular dysfunction may lead to a false sense of security and undertreatment. Consequently, the consistency between FFR and myocardial perfusion imaging is reduced. Lee et al.'s analysis of 313 patients with high FFR (> 0.80) further demonstrated that low coronary flow reserve and high microvascular resistance index independently predict adverse outcomes, highlighting microvascular pathology's prognostic significance even when normal FFR values 35.

The integration of FFR with imaging modalities enhances diagnostic precision 36. Future developments aim to establish hybrid protocols combining anatomical and functional data for comprehensive coronary evaluation.

### 3.2 Instantaneous Wave-Free Ratio (iFR)

Instantaneous wave-free ratio (iFR) measures the ratio of distal coronary to aortic pressure during the diastolic wave-free period. While normal coronary vessels maintain constant pressure gradients along their course, stenotic lesions induce pressure decay due to energy dissipation. iFR delivers intracoronary pressure measurements equivalent to pressure wire-acquired FFR, utilizing pressure wire measurements without requiring vasodilators. Notably, iFR measurement optimizes clinical workflows through reduced assessment duration while offering distinct advantages for adenosine-contraindicated patients, a demographic overrepresented among those presenting with ISR 37.

However, a significant research gap exists: these pivotal trials largely excluded or did not specifically analyze patients with ISR. Berry et al.'s multinational longitudinal study demonstrates weak iFR-FFR correlation (r = 0.32, p < 0.05), fundamentally challenging its clinical utility in stenosis management 38. The iFR framework, rooted in wave intensity analysis (WIA), defines quiescent phases, postulates pharmacotherapy-free vasodilation during diastolic pressure convergence. Experimental validation reveals iFR's inherent limitations as a microcirculatory resistance index, violating Ohm's law principles and demonstrating restricted stability across vasomotor states. While offering procedural efficiencies, iFR's clinical adoption remains limited by inadequate RCT validation and persistent hemodynamic modeling constraints. Current evidence for iFR in the specific context of restenotic lesions remains limited to small observational series, and long-term outcomes beyond five years for iFR-guided ISR management are entirely lacking. Conducting extended follow-up research is therefore imperative to comprehensively evaluate long-term outcomes, address this gap, and provide more robust clinical evidence.

## 4. Multimodal Imaging Integration for Diagnosis

### 4.1 CT-Derived FFR (CT-FFR)

Computed tomography-derived fractional flow reserve (CT-FFR) non-invasively calculates fractional flow reserve by applying computational fluid dynamics (CFD) and machine learning algorithms to CTA datasets 39. Approved for clinical use in multiple regions, CT-FFR serves as a critical adjunct for screening, diagnosis, and revascularization decision-making.

This technology addresses a critical unmet need in ISR management: the lack of a non-invasive, accurate method for routine surveillance. As a non-invasive technique integrating anatomical and functional evaluation, CT-FFR eliminates procedural risks associated with invasive FFR and adenosine contraindications, particularly benefiting high-risk populations. By incorporating CTA-derived plaque characteristics (calcification distribution, plaque burden) with hemodynamic data, it provides comprehensive insights for managing diffuse and tandem lesions 40. In post-PCI follow-up assessments, CT-FFR has demonstrated efficacy in identifying ISR and distal flow limitations, exhibiting high diagnostic concordance with invasive FFR measurements 41. Multicenter validation studies have validated diagnostic reliability, with CT-FFR exhibiting strong correlations with invasive FFR and demonstrating ischemia-specific specificity of 84% in non-calcified lesions 42-44. CT-FFR offers the potential for earlier detection of hemodynamically significant restenosis, enabling timely intervention before progression to myocardial infarction.

Despite its promise, the translation of CT-FFR into routine ISR surveillance faces several population-specific barriers. Current challenges include stringent CTA quality requirements and physiological variability. Optimized computational models and accelerated processing enable expanded applications, including emergency settings. Cost-effectiveness analyses indicate CT-FFR reduces unnecessary revascularizations 45. The PRECISE randomized trial first confirmed that a risk-stratified strategy based on CTA with selective FFR-CT can significantly optimize the treatment pathway for patients at high risk of ISR, reduce inefficient angioplasty, and identify high-risk ischemic lesions requiring intervention 46.

Technical constraints persist, particularly in severely calcified lesions, vessel tortuosity, and stent artifact interference (> 0.05 FFR deviation). Accuracy diminishes in small distal vessels. Future developments focus on refining CFD models through shear stress analysis and artifact suppression 47. AI-enhanced algorithms improve computational efficiency, while calcium correction and microvascular resistance modeling promise accuracy improvements 48. The development and external validation of ISR-dedicated prediction models incorporating serial CT-FFR metrics represent a critical unmet need.

Multifactorial predictive models integrating clinical data, imaging features, and biomarkers enable early detection of ISR. These models improve risk stratification accuracy through multimodal data synthesis. He et al. developed and validated a nomogram-based prediction model incorporating clinical variables including diabetes mellitus, lesion length, and stent diameter, achieving robust discrimination for ISR risk stratification 49. Similarly, Deng et al. reported that systemic immune-inflammation index, when integrated with angiographic features, significantly enhanced predictive performance for ISR compared to clinical factors alone 50. Multimodal data synthesis combining clinical, imaging, and biomarker information represents a promising direction for precision medicine in ISR management, enabling earlier identification of high-risk patients and more targeted therapeutic interventions.

### 4.2 Resting Full-Cycle Ratio (RFR)

The functional assessment of ISR by hyperemic pressure indices is frequently compromised by the high prevalence of adenosine contraindications, adenosine resistance, and microvascular dysfunction in this population. Resting Full-Cycle Ratio (RFR) is an emerging tool for the functional assessment of coronary arteries. It utilizes the ratio of distal coronary pressure (Pd) to aortic pressure (Pa) across the entire cardiac cycle, offers a theoretical solution to this clinical dilemma. Unlike traditional indices that focus solely on specific cardiac phases, RFR integrates hemodynamic information from both diastole and systole, providing comprehensive assessment of restenotic lesion severity without pharmacological stress 51. RFR demonstrates high diagnostic accuracy and excellent correlation with FFR 52,53, establishing its clinical validity. Notably, RFR exhibits robust discriminatory capacity, particularly in identifying hemodynamically significant stenoses potentially overlooked by conventional methods 54. However, despite its advantages over other resting indices, RFR remains susceptible to confounding factors. In cases of abnormal coronary microcirculation, the results may be subject to certain interferences 55.

### 4.3 Quantitative Flow Ratio (QFR)

Quantitative Flow Ratio (QFR), an angiography-derived FFR technology originating in China, calculates fractional flow reserve via three-dimensional vascular reconstruction and computational fluid dynamics analysis of coronary angiography images. For ISR surveillance, where repeated invasive functional assessment is often deferred due to procedural concerns, QFR offers a mechanism to integrate physiological evaluation into every follow-up angiogram without additional risk or cost. The 2024 European Guidelines for Chronic Coronary Syndrome (CCS) Management designate QFR as a Class I recommendation, recognizing its role as a novel standard for coronary functional evaluation 56. This guideline endorsement, while based primarily on *de novo* lesion data, establishes a framework for extending QFR to ISR assessment—a logical next step given the shared technical principles.

QFR demonstrates enhanced predictive performance for FFR assessment relative to conventional FFR and iFR techniques. This innovation derives its distinct procedural merits from obviating both intracoronary pressure wire deployment and pharmacological hyperemia induction, minimizing procedural complexity and patient discomfort while preserving diagnostic precision 57. The clinical application value of QFR requires further prospective validation, particularly regarding hard endpoints. Measurement reproducibility is influenced by angiographic quality, observer-dependent stenosis assessment, and reference FFR values 58. For ISR, these technical constraints are amplified by stent-related artifacts and the complex morphology of restenotic lesions, underscoring the need for ISR-specific validation studies.

By addressing key limitations of existing techniques, QFR exhibits heightened diagnostic performance, positioning it as a promising future standard for evaluating coronary physiological function 59. However, the FAVOR III Europe trial indicates QFR fails to demonstrate clinical outcome equivalence when invasive FFR is available. Conversely, evidence suggests QFR outperforms visual stenosis assessment in settings where FFR is inaccessible 60. As it is based on CAG, QFR is an invasive test with complex procedures and a longer duration. It also involves significant radiation and contrast agent use, which is contraindicated for some patients. Moreover, its accuracy is compromised when evaluating highly complex lesions. Whether QFR can be widely used in CAD phenotypes remains to be seen. Its broad clinical potential requires further study.

### 4.4 Optical Flow Ratio (OFR)

ISR is characterized by distinct morphological phenotypes—ranging from homogeneous neointimal hyperplasia to heterogeneous neoatherosclerosis with high-risk plaque features—that can be precisely delineated by OCT but lack direct correlation with hemodynamic significance 61. Optical flow ratio (OFR), an OCT-based innovation, rapidly computes FFR by integrating multidimensional OCT data. This evolution enables simultaneous assessment of plaque morphology and coronary physiology. It overcomes angiography limitations in lumen boundary detection—particularly calcium artifacts and contrast filling heterogeneity—thereby advancing integrated morpho-functional diagnostics 62. OFR's unique algorithmic architecture maintains diagnostic accuracy irrespective of stent implantation status, suggesting robustness to metallic artifact 63. This technical characteristic holds particular promise for ISR assessment, offering the potential to derive FFR-equivalent metrics without pressure wire traversal through restenotic tissue—avoiding theoretical risks of neointimal disruption and reducing procedural complexity.

Despite its advantages, OFR's clinical implementation faces critical barriers. Similar to early QFR development, multicenter randomized controlled trial evidence remains absent. Validation in high-risk plaque phenotypes and complex anatomies requires further investigation 64. Future research should establish causal relationships between OFR-guided revascularization and MACE reduction while exploring synergistic applications with intravascular ultrasound and determining whether serial OFR assessment can predict ISR progression or guide personalized surveillance intervals in stented patients.

### 4.5 Intravascular Ultrasound-Based Fractional Flow Reserve (UFR)

Intravascular Ultrasound-Based Fractional Flow Reserve (UFR) is a novel method integrating IVUS imaging to determine whether anatomical findings correspond to hemodynamically significant flow limitation. A retrospective single-center study by Yu et al. shows that due to its short analysis time, UFR analysis can immediately assess the physiological significance of coronary stenosis right after IVUS image acquisition without extra instruments 65. In the ISR context, this capability holds theoretical promise: it would enable operators to determine, during the same procedure, not only why the stent has restenosed (via IVUS) but also whether the restenosis is flow-limiting and requires intervention (via UFR).

Sui et al. compared UFR based on intravascular ultrasound with QFR based on angiography in diagnosing LMCA stenosis and showed that UFR has a strong correlation and good consistency with FFR 66. Current evidence highlights the value of UFR in minimizing over-treatment and facilitating prognosis stratification, particularly in diffuse lesions and calcified nodules. UFR accurately assesses stenosis significance, identifies specific segments requiring dilation in diffuse lesions, and evaluates the hemodynamic impact of calcified nodules to guide treatment strategies such as calcium modification. However, direct evidence for UFR in ISR remains entirely absent, as validation studies explicitly excluded patients with prior stents. Consequently, the diagnostic accuracy, optimal thresholds, and prognostic value of UFR in restenotic lesions are completely unknown.

### 4.6 CTA-OCT/IVUS Integration

ISR represents a distinct disease entity characterized by in-stent neointimal proliferation and neoatherosclerosis, whose comprehensive evaluation requires integration of non-invasive detection, invasive mechanistic diagnosis, and risk stratification—a paradigm that mirrors the broader trend toward multimodal plaque characterization. While CTA provides detailed anatomical information about complex lesions, it lacks direct functional assessment. Intravascular imaging modalities (OCT/IVUS) enable precise intraprocedural evaluation for stent optimization. Growing evidence suggests no single technique comprehensively evaluates coronary atherosclerosis. Multimodal integration synthesizes morphological and compositional data, advancing the understanding of acute coronary syndrome (ACS) pathophysiology and guiding targeted therapies 67.

Voros et al., based on differences in CT attenuation values, highlighted the association between the imaging characteristics of non-calcified plaques and their potential histological composition, establishing a foundation for CTA as an effective non-invasive tool for plaque characterization 68. Furthermore, CTA surpasses CAG by enabling three-dimensional plaque characterization and identification of high-risk features. This positions CTA as an effective gatekeeper for invasive imaging, streamlining workflow through targeted patient selection, which reduces unnecessary procedures and focuses valuable resources on suspicious and complex lesions.

CTA and intravascular imaging (IVUS/OCT) serve complementary roles: CTA delivers a macroscopic stent overview, whereas IVUS/OCT provides microscopic structural details. Athanasiou L et al. confirmed a strong correlation between the luminal, outer vessel wall, and volumetric parameters obtained from IVUS and CTA, thereby validating the reliability of CTA-based models for non-invasive vascular assessment 69. Cao et al. demonstrated the excellent diagnostic performance of CTA in detecting coronary plaque and accurately identifying its type, underscoring its significant value for risk stratification 70.

### 4.7 IVUS-OCT Dual-Modality Catheter

Multimodal imaging techniques have shown significant clinical potential for ISR diagnosis, their core strength lies in integrating complementary data from distinct modalities to overcome limitations inherent in single-technique approaches. IVUS excels at detecting mechanical abnormalities—stent underexpansion, fracture, edge dissection—while OCT provides unparalleled resolution for characterizing in-stent tissue—neointimal patterns, neoatherosclerosis, thrombus, and uncovered struts. Through technical complementarity and data synergy, multimodal imaging enhances diagnostic accuracy and treatment monitoring, particularly in complex lesion management 71.

However, the high cost of the dual-modality IVUS-OCT system, along with its technical complexity and operational difficulty, somewhat limits its widespread application, making it challenging to implement in regions with limited medical resources. While the two systems are functionally complementary, there is a conflict between resolution and penetration depth. OCT offers high resolution but is limited in imaging depth, whereas IVUS has strong penetration but relatively low resolution, which may affect the comprehensive evaluation of certain lesions 72. Furthermore, signal interference and artifacts can impact image quality and diagnostic accuracy, and currently, there is a lack of unified diagnostic standards.

AI-powered analysis of dual-modality imaging holds particular promise. Advancements in AI and miniaturization technologies position multimodal imaging as a cornerstone for precision coronary intervention, heralding an era of intelligent personalized cardiovascular care 73.

## 5. Biomarker Analysis

There have been significant advances in the study of molecular mechanisms of ISR. Biomolecular markers attract considerable research interest due to their potential in risk stratification, early diagnosis, and prediction of therapeutic efficacy. It is crucial to search for innovative therapeutic targets based on the pathogenesis.

Future ISR diagnosis will pivot toward the convergence of bioengineering, data science, and clinical medicine. This integration will advance management beyond traditional imaging and empirical approaches, enabling multidimensional, dynamic, and personalized strategies.

### 5.1 Inflammation-Related Markers

As a classic marker of inflammation, C-reactive protein (CRP) has been proven to be related to ISR risk in numerous studies 74,75. Elevated plasma levels not only reflect the systemic inflammatory state but also promote vascular smooth muscle cell proliferation and neo-intimal formation by activating the NF-κB pathway in endothelial cells, suggesting its potential as an indicator of atherosclerosis progression 76. Additional inflammatory mediators similarly predict ISR occurrence. Redox processes exacerbate the inflammatory response through the generation of reactive oxygen species (ROS) and reactive nitrogen species (RNS), leading to vascular smooth muscle cell proliferation and migration, thereby promoting ISR development. Inflammation and oxidative stress interact, creating a vicious cycle that drives the pathological process of ISR 77. The neutrophil-to-lymphocyte ratio (NLR) elevation independently predicts ISR risk across diverse vascular conditions 78, with inflammation and oxidative stress forming a self-perpetuating pathological cycle.

### 5.2 Endothelial Dysfunction-Related Markers

Non-invasive endothelial function testing identifies endothelial dysfunction as a predictor of late ISR progression, offering diagnostic and prognostic utility 79. Integration of genetic data on intimal hyperplasia-associated variants facilitates risk stratification and personalized monitoring. Bioinformatics analyses have further identified endothelial dysfunction-related genes as potential therapeutic targets and diagnostic biomarkers for atherosclerotic risk assessment, providing supplementary ISR diagnostic support 80.

### 5.3 Genomic and Transcriptomic Markers

High-throughput sequencing reveals genetic susceptibility to ISR, with genome-wide association studies (GWAS) identifying multiple associated loci 81. High-throughput sequencing enables patient-specific genetic risk score (GRS) construction to predict ISR probability, offering genetic adjuncts for diagnosis 82. Additionally, AI-driven multimodal models that integrate clinical data, imaging features, and GRS can significantly enhance the accuracy of ISR risk prediction 82.

### 5.4 Emerging Therapeutic Targets and Marker-Target Integration

Some drug target-related molecules also have the potential to be biomarkers. Future large-scale prospective cohort studies should validate marker efficacy while exploring epigenetic regulation and endothelial-immune cell interactions in ISR pathogenesis. This approach will advance precision medicine through individualized diagnostic and therapeutic strategies.

## 6. Emerging Technologies

### 6.1 Photon-Counting CT (PCCT)

Photon-counting CT (PCCT), an emerging CT technology based on energy-resolving, direct-conversion X-ray detectors, captures the energy information of individual X-ray photons via photon-counting detectors (PCDs), achieving unprecedented spatial resolution (150-200 μm) with pixel sizes of 0.1-0.2 mm 83. With its ultra-high resolution, multi-energy imaging, and low radiation dose characteristics, PCCT offers a new perspective for ISR diagnosis and mechanism research. By analyzing X-ray photon energy spectra, it differentiates material compositions precisely 26. Its spectral imaging capability classifies photons into distinct energy bins, enabling advanced reconstruction techniques such as virtual non-iodine (VNI) and virtual monoenergetic images (VMI) 84. PCCT demonstrates superior ISR diagnostic performance by enhancing spatial and contrast resolution with reduced noise and artifacts. It significantly decreases radiation exposure and contrast volume during CTA, particularly improving stent lumen visualization in severe calcification 85. Combined with CT-FFR, PCCT enhances functional ISR assessment while minimizing invasive procedures for high-risk patients.

Leveraging multi-energy parametric imaging, PCCT discriminates materials by atomic number 86. Beyond quantifying plaque burden, it identifies neointimal fibrous and lipid components and enables AI-driven volumetric measurements, informing intervention strategies.

Despite its theoretical advantages for non-invasive ISR surveillance, PCCT faces several challenges that currently limit its evidence-based application in this population, corresponding to specific knowledge gap. Although metal artifacts are reduced, high-atomic-number stents may still impair peri-stent imaging 87. Current PCCT-ISR studies are limited by small sample sizes, requiring large-scale prospective validation.

### 6.2 Deep Learning-Based Reconstruction (DLR): AiCE System

Non-invasive ISR surveillance by CT has been historically limited by three interconnected challenges: cumulative radiation exposure from repeated imaging, image noise compromising detection of subtle in-stent changes, and metallic stent artifacts obscuring lumen visualization. The integration of machine learning (ML) addresses these challenges simultaneously, enhancing standardization across the ISR imaging chain. Its high accuracy and efficiency are crucial for clinical practice. The system aids physicians in more accurately diagnosing and treating stable angina patients, reduces unnecessary invasive procedures, and improves patient acceptance and early diagnosis feasibility 88.

For ISR, where even small improvements in diagnostic confidence can prevent unnecessary repeat catheterizations, AI-enhanced imaging offers a pathway to safer, more efficient diagnosis. The AI-powered AiCE system utilizes convolutional neural networks (CNNs) trained on high-resolution medical images to restore diagnostic-quality images from low-dose or noisy data. Using deep convolutional neural networks (DCNNs), AiCE learns features from model-based iterative reconstruction (MBIR) images to enhance hybrid iterative reconstruction (HIR) outputs, achieving MBIR-equivalent quality with reduced computational demands 89.

This technology distinguishes true signals from noise without increasing radiation, preserving anatomical fidelity critical for longitudinal comparison of in-stent luminal dimensions and early detection of restenosis progression. Integrated into CT workflows, AiCE delivers rapid, high-definition imaging. Recent developments apply deep learning to mitigate metal artifacts, outperforming conventional methods in primary and secondary artifact suppression 90.

AI model performance critically depends on training data diversity. Limited demographic or equipment representation in training datasets may compromise generalizability and artifact correction stability. High-quality, multi-source annotated datasets are essential for algorithm robustness 91. Deep learning reconstruction represents a paradigm shift for CT, MR, and PET imaging. For ISR in particular, it provides the technological foundation for a future in which surveillance is personalized, radiation-minimized, and invasively confirmed only when AI-flagged abnormalities warrant catheterization.

## 7. Clinical Strategies and Discussion

ISR assessment requires combining anatomical, imaging, and functional data for a complete evaluation. CAG is the preferred initial diagnostic method due to its ease of access and quick results. Despite its capabilities, the method has limitations in identifying mild stenosis and struggles to accurately detect early signs of ISR. OCT and IVUS offer detailed vascular imaging with high-resolution images. These imaging techniques can identify neointimal hyperplasia and detect stent-related mechanical issues, providing crucial insights for treatment decisions.

Functional validation via methods like FFR and iFR is critical for moderate stenosis management. These assessments determine stenosis hemodynamic significance, guide treatment decisions, and ensure only functionally significant lesions are treated. For high-risk individuals, regular monitoring of inflammatory biomarkers and imaging helps to provide a more complete picture. This method enables early identification of ISR and facilitates adjustments to treatment plans to prevent serious complications. Multimodal imaging integration has revolutionized ISR diagnosis. CT-FFR, QFR, OFR, UFR, and RFR offer unique benefits. The combined approach provides a complete assessment of ISR, encompassing both anatomical and functional aspects. This multi-dimensional approach enhances early ISR detection, supports lesion-specific treatment, improves outcomes, and advances precision medicine in interventional cardiology.

**Figure 1:**
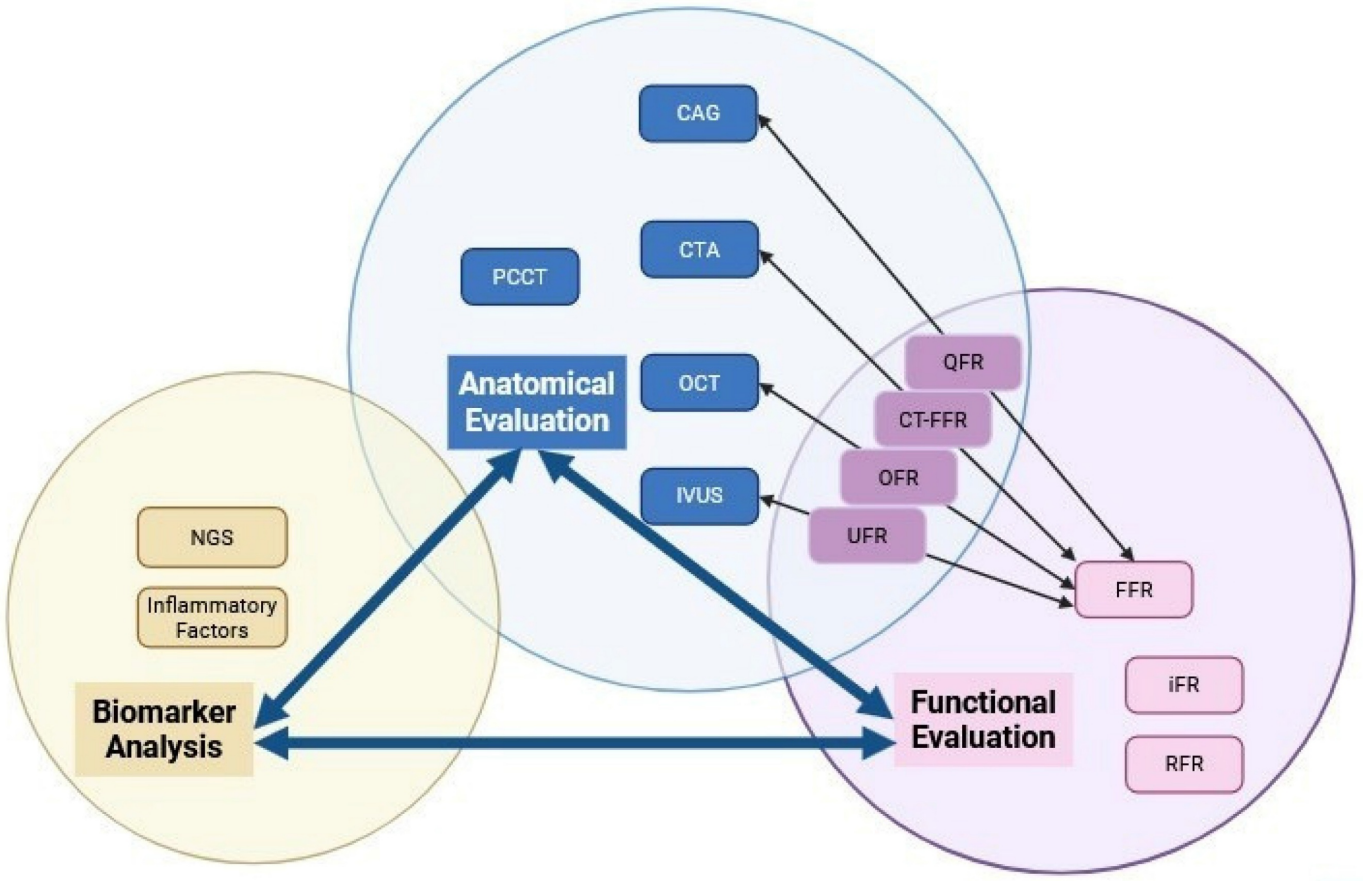
Categorization and Relationships Among Imaging Modalities. The diagram schematic illustrates the relationships of three primary evaluation domains: anatomical evaluation, functional evaluation, and biomarker analysis. It explicitly frames these as an interconnected diagnostic cascade, where each domain both informs and complements the others to enable comprehensive assessment.

The evolution of ISR diagnosis has shifted from traditional anatomical assessment to sophisticated multimodal functional evaluation. The shift towards improved ISR detection and subtype classification is fueled by advancements in technologies such as PCCT, AI, and novel biomarkers. AI integration with multimodal imaging analysis is becoming central to ISR diagnosis. AI algorithms can analyze vast data from different imaging modalities and clinical parameters, identifying patterns and correlations. Advanced technologies and AI-powered multimodal analysis create effective and accurate diagnostic tools. By utilizing these frameworks, clinicians can conduct thorough evaluations, leading to improved patient outcomes through early intervention and tailored treatment plans. A schematic conclusion ofthe relationships in between three primary evaluation domains can be found in Figure 1.

## Figures and Tables

**Table 1:** Comparative analysis of coronary imaging technologies in the Context of ISR

	CAG	CTA	OCT	IVUS	VH-IVUS	IB-IVUS	ADE	NIRS	PCCT
Principle	X-ray imaging	CT technology 3D images	Infrared light interference imaging	HF-US and radiofrequency RF spectrum analysis	Color coding	Backscatter	Automated Differential Echogenicity	Near-infrared light absorption	X-ray imaging
Resolution	Relatively low	400-600 microns 3,4	10 - 20 microns 21	100 - 150 microns 11					Superior spatial resolution 26
Penetration Depth	Capable of penetrating the entire vascular lumen		1 - 2 millimeters	4 - 8 millimeters 11					Moderate
Imaging Speed	Fast	Moderate	Fast	Slow, affecting imaging efficiency				Fast	Ultra-fast
Advantages	Accurate	Non-invasive	High resolution	Comprehensive vascular assessment and remodeling Assess stent expansion and malapposition					Reduced overestimation of calcification volume
Characteristics	2D	3D	Allowing more precise differentiation of vascular wall layers	Accurate evaluation of stent apposition and external compression	Identifying vulnerable and lipid-rich plaques	Enhanced discrimination between lipid-laden atherosclerotic lesions and high-risk vulnerable plaques	Mitigates the subjective bias characteristic of manual analytical approaches	Quantitative profiling of molecular constituents in endothelial layers	Enhanced visualization of intravascular microstructures and lesions
Clinical Application	Preliminary assessment Guidance for interventional procedures	Initial vascular assessment Guidance for stent implantation	Detection of TCFA Risk stratification Visualization of thin-layer intimal hyperplasia Prediction of stent under-expansion	Complex lesions long-segment vascular imaging left main coronary artery lesions				Morphological assessment of in-stent neointimal proliferation, intimal dissection, and thrombogenic activity	Detailed imaging of local lesions Simultaneous multi-coronary artery imaging Comprehensive observation
Limitations	Unable to show vessel wall details	Lower accuracy than CAG	Shallow imaging depth Susceptible to blood interference Inability to perform prolonged measurements	Constrained resolution Depends on operator's experience Restricted detection sensitivity for microstructural pathological alterations					Existence of noise interference Technically demanding High maintenance costs

**Table 2:** Comparative analysis of coronary multimodal functional technologies in the Context of ISR

	FFR	CT-FFR	QFR	OFR	UFR	RFR
**Principle**	Measure the pressure ratio between the distal end of the lesion and the aorta	Based on CTA Computational fluid dynamics 3D model	Based on CAG Computational fluid dynamics 3D model	Based on OCT High-resolution intraluminal information	Based on IVUS Intraluminal information	Ratio of distal coronary pressure to aortic pressure
**Accuracy**	Gold standard with high diagnostic accuracy, over 80% 35	In accordance with FFR	High diagnostic accuracy, over 90% 58 Good correlation and consistency with FFR	High diagnostic accuracy, over 90% 62 Good correlation and consistency with FFR	High diagnostic accuracy, over 90% 66 Good correlation and consistency with FFR	Good correlation and consistency with FFR 52,53
**Convenience**	Require drug induction and pressure-guided wire	Non-invasive Rapid No additional equipment or consumables are required	Require pressure-guided wire	Require dedicated equipment and specialized software for calculation	Require dedicated equipment and specialized software for calculation	No need for vasodilators
**Invasive**	√	×	√	×	×	√
**Drug induction**	√	×	×	×	×	×
**Application scope**	Assessment of coronary artery stenosis lesions Identify hemodynamic significance of ISR	Assessment of coronary artery stenosis lesions and plaque status Mainly used for screening and initial evaluation	Formulate revascularization strategies Assessment of bifurcation and diffuse lesions	Auxiliary tool for coronary artery intervention Facilitate assessment of stenting outcomes	Assessment of severe calcified lesions Reduce target lesion revascularization Assist in formulating interventional treatment strategies and long-term prognosis for left main coronary bifurcation lesions	Identify potentially overlooked lesions
**Limitations**	Require maximum hyperemia induction, which some patients tolerate poorly	Different algorithms vary in accuracy and reliability	Relatively high cost easily affected by intravascular blood, increasing computational complexity and error Less accurate than OFR and UFR in complex lesions Need large-scale studies	Relatively high cost Easily influenced by intravascular blood Need large-scale studies	Relatively high cost Depending on the operator's experience Less sensitive to fully reflecting individual hemodynamic differences Need large-scale studies	Susceptible to the influence of abnormal circulatory function Need large-scale studies

**Table 3:** Summary of Major Large-Scale Trials and Key Evidence

Study	Study type	Results	Clinical Significance
**IVUS-XPL** **(IVUS) 10**	Randomized Clinical Trial	MACE incidence: 2.9% (IVUS-guided) vs. 5.8% (angiography-guided), P=0.007 TLR: 2.5% (IVUS-guided) vs. 5.0% (angiography-guided); HR 0.51, P=0.02 Cardiac death and target lesion-related MI were comparable between groups. Post-dilation was performed more frequently (76% vs. 57%), resulting in a larger final minimum lumen diameter in the IVUS-guided group.	Demonstrates superiority of IVUS-guided over angiography-guided second generation DES implantation in long lesions Provides high-level evidence supporting IVUS use in complex coronary intervention. Suggests IVUS reduces TLR risk by optimizing stent expansion and luminal gain. Supports routine IVUS use in long lesions to improve clinical outcomes.
**iOPEN** **(IVUS) 11**	Single-center, observational, retrospective cohort study	MACE: 18.0% (IVUS-guided) vs. 24.5% (angiography-guided), p=0.0014 TLR: 14.5% (IVUS-guided) vs. 19.2% (angiography-guided); p=0.021 More frequent post-dilation (18.6% vs. 14.1%) Larger stent diameter (3.04 mm vs. 2.94 mm) in IVUS group Shorter hospitalization (2.1 vs. 2.5 days) with IVUS, despite longer procedure time and contrast volume	Provides large-scale real-world evidence for IVUS use in ISR-PCI IVUS guidance significantly reduces 1-year MACE risk in ISR patients Supports routine IVUS in ISR to identify failure mechanisms & optimize strategy
**PRESTIGE** **(OCT) 19**	Multicenter, prospective, observational registry	DES implantation: Significantly associated with neoatherosclerosis, p=0.02	First large-scale prospective registry linking DES implantation with accelerated neoatherosclerosisSupports OCT's clinical utility for identifying high-risk plaque features Highlights need for vigilant monitoring of neoatherosclerosis in patients with prior MI
**PRECISE** **(CT-FFR) 46**	Multicenter, prospective, randomized controlled trial	Primary Endpoint (Efficiency + Safety): 4.2% (Precision) vs. 11.3% (Usual), HR 0.35 Procedural Efficiency: Lower catheterization rate without obstructive CAD (2.6% vs. 10.2%) Safety: No significant difference in death/nonfatal MI Medical Therapy Optimization: Higher 1-year use of lipid-lowering (50.0% vs. 41.8%) and antiplatelet (35.7% vs. 27.1%) drugs in Precision group Diagnostic Precision: Fewer total tests, higher initial test positivity (18.3% vs. 13.3%), and higher diagnostic yield of obstructive CAD at catheterization (80.0% vs. 39.5%)	First large RCT demonstrating that a precision strategy using quantitative risk stratification (PMRS) with CTA/selective FFR-CT improves efficiency and reduces unnecessary procedures. Provides RCT evidence for risk-based testing and offers an actionable protocol. Increases diagnostic yield of catheterization and optimizes medical therapy without compromising safety.
**VALIDATE** **(iFR) 52**	Retrospective, multicenter, diagnostic study	High Concordance: RFR highly correlated with iFR, R²=0.99 Equivalent Diagnostic Performance: Accuracy 97.4% vs. iFR Cycle Phase Independence: Minimum Pd/Pa occurred outside diastole in 12.2% of cycles (32.4% in RCA). Statistical Diagnostic Equivalence: Met predefined equivalence criteria, p=0.03	Represents a potentially superior tool for non-hyperemic physiologic assessment with clinical translation potential
**FAVOR III** **(QFR) 60**	Multicenter, randomized, sham-controlled, blinded clinical trial	1-year MACE incidence: 5.8% (QFR-guided) vs. 8.8% (angiography-guided), P=0.0004 Lower rates of MI (3.4% vs 5.7%) and ischemia-driven revascularization (2.0% vs 3.1%). Procedural Impact: QFR altered initial treatment strategy in 23.3% of patients, leading to fewer stents, less contrast, and shorter procedure time. Revascularization Quality: Higher rate of functional complete revascularization (88.1% vs 82.2%).	First large-scale RCT demonstrating that wire-free, angiography-based physiology (QFR) improves clinical outcomes post-PCI Offers a convenient, adenosine- and wire-free alternative to increase adoption of physiological assessment. Improves outcomes while reducing resource use (stents, contrast, radiation), indicating health economic benefits. Advances the shift from anatomy-guided to function-guided intervention, supporting QFR as a new standard of care.

## Abbreviations

ISR: In-stent Restenosis
PCI: Percutaneous Coronary Intervention
MACE: Major Adverse Cardiovascular Events
CAG: Coronary Angiography
CTA: Coronary Computed Tomography Angiography
OCT: Optical Coherence Tomography
IVUS: Intravascular Ultrasound
VH-IVUS: Virtual Histology Intravascular Ultrasound
IB-IVUS: Integrated Backscatter Intravascular Ultrasound
ADE: Automated Differential Echogenicity
NIRS: Near-Infrared Spectroscopy
PCCT: Photon-Counting CT
TCFA: thin-cap fibroatheroma
FFR: Fractional Flow Reserve
CT-FFR: Computed Tomography-derived Fractional Flow Reserve
iFR: instantaneous wave-free ratio
QFR: Quantitative Flow Ratio
OFR: Optical Flow Ratio
UFR: Intravascular Ultrasound-Based Fractional Flow Reserve
RFR: Resting Full-Cycle Ratio
AI: Artificial Intelligence
RCT: Randomized Controlled Trial
LMCA: Left Main Coronary Artery
CRP: C-Reactive Protein
PCCT: Photon-Counting CT
NGS: Next-Generation Sequencing
